# Central composite design driven optimization of sustainable stability indicating HPLC method for the determination of Tigecycline and greenness assessment

**DOI:** 10.12688/f1000research.130861.2

**Published:** 2023-08-10

**Authors:** Hani Mohammed Hafez, Sona Soliman Barghash, Marwa M. Soliman, Moustafa K. Soltan, Mohamed Abd Elrahman, Noha Salah Katamesh

**Affiliations:** 1 Pharmaceutical Chemistry Department, College of Pharmacy, Al-Esraa University, Baghdad, 10045, Iraq; 2 Pharmaceutical Analytical Chemistry Department, Faculty of Pharmacy (Girls), Al-Azhar University, Cairo, Egypt; 3 Pharmacy Department, Oman College of health sciences, Muscat, Muscat, Oman; 4 Medicinal Chemistry Department, Faculty of Pharmacy, Zagazig University, Zagazig, Sharqia, Egypt; 5 Department of Pharmacy, Al-Mustaqbal University College, Babylon, Iraq

**Keywords:** Tigecycline; Green liquid chromatography; GAPI; Stability indicating; HPLC; experimental design

## Abstract

**Background:** Tigecycline (TGC) is a recently developed antibiotic to battle resistant bacteria. The procedures outlined in the literature for analyzing TGC involve chemical solvents that could be hazardous. Therefore, this study aimed to create a sustainable and stable HPLC technique for quantifying Tigecycline in lyophilized powder. The powerful chemometric tool, experimental design (ED), will be applied to analyze the variables' interaction and impact on the selected analytical target profiles. Response surface methodology provides a tutorial on using the central composite design with three levels of variables and quadratic programming to optimize the design space of the developed method.

**Methods:** The New HPLC method consisted of an aqueous buffer and ethanol as a green mobile phase run on a reversed-phase symmetry C18 column. A full resolution between the Tigecycline and its degradation product peaks was achieved in a short analytical runtime.

**Results:** Further, the specificity, accuracy, precision, robustness and stability indicating power of the proposed approach were verified through stress degrading testing.

**Conclusions:** Finally, the analytical eco-scale and the green Analytical Procedure Index (GAPI) were utilized to determine how environmentally friendly the recommended method was compared to other published approaches.

## Introduction

Tigecycline (TGC) is a commonly used antibiotic for treating bacteria resistant to other antibiotics. TGC was the first antibacterial agent of the glycylcycline family, and it has shown some promise as a treatment for those afflicted with infections that are resistant to other methods. ^1^^,^^2^ It was created due to the rising prevalence of antibiotic-resistant pathogens, including *S. aureus*, Acinetobacter, and *E. coli*. Due to its structural modifications, its therapeutic action has been widened to encompass both +ve and –ve gram bacteria and types resistant to several drugs. ^3^

**Figure 1.**
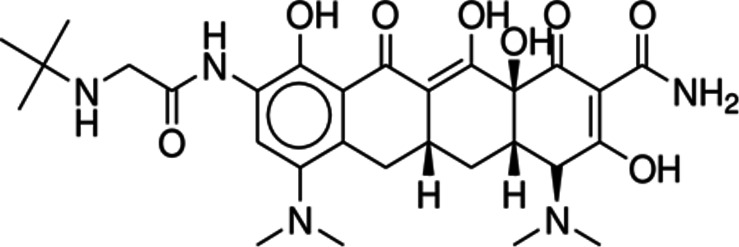
Chemical structure of Tigecycline.

Additionally, it has previously been examined by research participants in clinical studies as a single-drug therapy for treating bacterial infections that are difficult to treat. Indeed, its pharmacodynamics and pharmacokinetics aspects all play a role in this. Studies have indicated that TGC is more effective in treating complicated intra-abdominal and severe skin infections. TGC can only be obtained through intravenous administration. ^4^ TGC is a chemical molecule partially synthesized and obtained through fermentation. Because it possesses polar groups such as hydroxyl, amide, and amine groups, it is a tetracyclic molecule with a high degree of polarity, as shown in Figure 1 (which depicts the molecule's chemical structure). The chemical formula C _29_H _39_N _5_O _8_ has a molecular weight 585.65 ( Figure 1). TGC is freely soluble in water and slightly soluble in alcohol. ^5^^–^^7^

Before a pharmaceutical product may be created and marketed, the chemical stability of an active pharmaceutical substance must be proven. The International Conference on Harmonization mandates that recent pharmaceutical components and dosage forms undergo stability testing. ^8^^–^^10^ Investigation is required to understand the intrinsic stability qualities of the active substance in which hydrolysis is a potential issue. Acidic and basic hydrolyses are the most commonly used tests. ^9^^,^^10^ TGC was previously determined using spectrophotometric methods, ^11^ fluorescence determination, ^12^^–^^14^ and liquid chromatography coupled with ultraviolet (HPLC-UV) ^15^ and mass spectrometry detection in human plasma (LC-MS-MS) ^16^^–^^26^ and pharmaceutical preparations. ^27^^–^^32^ Other methods, such as stability-indicating, ^33^^–^^40^ have also been used to determine the drug's parenteral dosage form. These analytical methods take significant time, are harmful to the surrounding ecosystem, and require using acetonitrile as a mobile phase.

Green analytical chemistry (GAC) has recently become a focus of attention in the scientific community. GAC is a subfield of analytical chemistry that aims to eliminate harmful materials' application in analytical techniques and reduce pollution. The amount of energy used and the amount of waste produced can be reduced ^41^^,^^42^ without impacting the analytical performance of the method. Consequently, this study focused on substituting less dangerous and environmentally friendly solvents for existing mobile phases to provide eco-friendly solutions first. TGC determination may be accomplished using simple and rapid chromatographic techniques that do not need sample extraction, pre-treatment filtration, or derivatization. The (GAPI), the Green Analytical Procedure Index, and the Eco-scale analytical tool were used to assess the chromatographic method's environmental friendliness. ^43^^,^^44^ In addition, tests of drug products' chemical stability are subjected to artificially induced degradation to evaluate the results of these tests.

HPLC is a dynamic separation technology with many applications, but the procedure is ultra-critical because so many parameters must be modified before each run. Therefore, these methods need more understanding. For example, the experimental design (ED) approach was utilized to save the time and effort required to reduce the experiment's number, reagent consumption, and laboratory work. ED identifies and quantifies links between factors and responses. Furthermore, ED explores how several factors affect response levels. ^45^^,^^46^ All significant variables and levels were examined in a response surface methodology (RSM) and central composite design (CCD). This design comprises a central core of a two-level factorial design (2 ^n^). In its role, this core consists of 2 ^n^ outer points and one center point. The application of ANOVA allows for the computation of the significance of the models' coefficients of studied variables. ^46^^,^^47^

Finally, to accomplish this goal, researchers have developed a stability-indicating HPLC approach that is environmentally friendly, easy, conventional, and sensitive, utilizing the advantages of ED methodology. This method is used to measure degradation products in TGC dosage form simultaneously. The approach was validated by adhering to the International Conference on Harmonization (ICH) standards. ^48^^,^^49^

## Methods

### Reagents and chemicals

The Quality for pharmaceutical services (QPS) laboratories (Egypt) donated TGC. Ammonium acetate, triethylamine (TEA), ammonium hydroxide, ethylene diamine tetra-acetic acid disodium salt dihydrate (EDTA), and acetic acid were obtained from Merck (Germany). Without additional purification, HPLC-grade ethanol from Merck (Germany) was utilized. A Milli-pore analytical deionization system collected deionized water (Bedford, MA). Tygacil ^®^ (Pfizer, U.S.A.) lyophilized vial containing 50 mg of TGC was purchased from wholesale suppliers during the product's shelf life.

### Instrumentation

A quaternary pump and a solvent chamber with an auto-sampler injection system were both parts of the Alliance HPLC instrumentation (Waters, U.S.A.) used in this study. A conventional flow cell was coupled to a Waters photodiode array for detection. The data was gathered with the help of the Empower 3 program. All results were calculated in Design Expert 13 software from Stat-Ease Inc. (Minneapolis, U.S.A.) and Microsoft Excel 2016 (Microsoft Corporation, U.S.A.). Lab pH meter model AD1030 from ADWA in Romania was used to adjust the mobile phase's pH to the desired level. The accurate weighing was accomplished using an analytical balance of SA 210 D (Scientech, U.S.A.).

### Chromatographic conditions

***Preparing an ammonium acetate pH 6.0 buffer***

Transfer 3.85 g ammonium acetate (50 mM), 5.82 g EDTA (20 mM), and 0.2 mL TEA (0.2% v:v) to 1000 mL HPLC grade water, sonicated to dissolve, and then adjust to pH 6.25with glacial acetic acid.

***Optimized chromatographic conditions***

TGC was assessed in pharmaceutical drug products and degradation investigations on a reversed-phase symmetry C18 column (10* 0.46 cm, 3.5 μm), Waters (Ireland). A mobile phase of buffer solution (50 mM ammonium acetate, 20 mM disodium edetate, 0.2% triethylamine) and ethanol 85:15 (v:v) was used for the liquid chromatography. The column thermostat was maintained at 40°C. It was filtered with a membrane filter from a Millipore of 0.45-μm pore size. The analysis was carried out at a flow rate of 1.0 mL per minute utilizing UV detection at 275 nm. The reference material and the samples were injected with a volume of 40 μL.

### Preparing reference substance solutions

The standard stock solution was produced by accurately weighing 25 mg of the TGC reference substance, moving it to a 25 mL flask, and diluting it with distilled water until it reached a TGC concentration of 1000 μg mL ^-1^. The freshly prepared stock standard solution was then diluted with water to the desired concentration before being filtered through a membrane filter with a pore size of 0.45 μm (Millipore).

### Construction of calibration graphs

Aliquots were taken from TGC standard stock solutions and transferred into appropriate volumetric flasks to prepare concentrations of 8-60 μg mL ^-1^ for TGC to complete each flask, and distilled water was utilized. The calibration graphs were created by graphing the area under the peak versus TGC concentration in μg mL ^-1^ and calculating the regression equations.

### Accuracy and Precision tests

The stock solution should be diluted for the precision test to produce solutions containing 32, 40, and 48 μg mL ^-1^. These solutions will be evaluated using three separate measurements of TGC samples on the same day (intra-day). Then, it was performed over two more days to assess the investigation's intermediate precision than initially planned (inter-day). For the objective of the accuracy test, exactly known quantities of TGC were successively transferred to a placebo solution to generate solutions with concentration levels of 32, 40, and 48 μg mL ^-1^, equivalent to 80, 100, and 120% of the actual analytical level.

### The preparation of test samples

To make the sample solution, weighing and mixing Tygacil ^®^ vials with 50 mg of TGC in each 25 mL flask was necessary. A suitable volume of the solution was added and mixed to the volume with distilled water (40 μg mL ^-1^) and injected.

### Forced degradation tests of TGC

A standard reference solution and pharmaceutical preparations of 1 mg mL ^-1^ concentration were submitted to rapid forced degradation under acidic, basic, thermal, oxidative, and photolytic conditions to study the interference in the measurement of TGC. The ICH guidelines Q1A (R2) and Q1B were used for these degradation investigations, including solid and solution phases. ^5^^,^^10^^,^^48^

***Acidic forced degradation***

10 mL of the TGC stock solution was treated with 100 mM HCl to hasten acidic degradation. Subsequently, the solution was examined at 60°C for an hour. ^5^^,^^10^

***Alkaline forced degradation***

To initiate alkaline degradation, a portion of 10 mL of TGC stock solution was exposed to 10 mL of 100 mM sodium hydroxide at 25°C for 2 hours. ^5^^,^^10^

***Oxidative forced degradation***

To commence the oxidative degradation, a portion of 10 mL of TGC stock solution was maintained at 25°C for two hours, shielded from illumination, with 10 mL of 5% H _2_O _2._^5^^,^^10^

***Thermal forced degradation***

TGC powder was thermally investigated and maintained at 60°C in a thermostated oven. A portion of the treated material was weighed and diluted in a 25-mL flask. ^5^^,^^10^

***Photolytic forced degradation***

TGC powder and solution were tested for photolytic degeneration after twenty-four hours of exposure to near-ultraviolet radiation. There was enough TGC powder weighed and tested under ultraviolet radiation. In addition, an amount of TGC stock solution equal to 10 mL of distilled water was treated with ultraviolet light for testing. ^5^^,^^10^^,^^48^ Therefore, every sample was taken at the specified intervals. If necessary, it was treated before being injected to stop the degradation, and then it was diluted with distilled water to the target concentration (40 μg mL ^-1^).

### Experimental design (ED)

Initially, a trial and error approach was used to learn about the method's efficacy and to identify critical, independent parameters and their impacts on dependent responses or parameters. Establishing a separation between TGC and its degradants with a resolution of more than 2.0 is the primary goal of the RP-HPLC method development, in addition to other important parameters that affect the accuracy and precision of the method. For example, the ED of the proposed method was set, and the central composite design (CCD) with the response surface method (RSM) was used. ^47^

The ED initially begins with the pre-determination of the principle parameter crucial to the method efficacy; therefore, it should be measured during the ED (critical quality attributes (CQA)). The next step was determining the acceptable value of the CQA according to universal pharmacopeias such as USP and BP, defined as analytical target profile (ATP). The optimum resolution (Rs) between peaks is considered the principle CQA. The peak symmetry, capacity factor, and other CQAs were also investigated ( Table 1). The proposed ATPs had to be determined ( Table 1) to achieve good reliability of the results. The next step is to define the main variables of the proposed chromatographic method that affect the CQAs and ATPs values which are defined as critical method parameters (CMPs) ( Table 1). ^45^

**Table 1.** CMP, CQA, and ATP of the proposed method.

CMP	The range for each parameter used for		CQA	ATP
	Low	High		Targeted ATP
**% Ethanol**	10	20	Resolution (Res 1) between Deg 1 and Deg 2	NLT 2
**Mobile phase pH**	5	7.5	Resolution (Res 2) between Deg 2 and TGC	NLT 2
**% EDTA**	0.01	0.04	Theoretical plates (T plate)	2000-4000
**% TEA**	0.1	0.3	Symmetry factor (sym)	0.8-1.2
			Capacity factor (K factor)	2-10
			Run time	NMT 6

Furthermore, the RSM with a three-level CCD was selected for the ED. In other words, the design comprises 3 important factors independently acting to build the design. The independent factors investigated were the ratio of green solvent (% Ethanol), the pH value of aqueous mobile phase (pH), and the concentration of additives EDTA and TEA.

Twenty runs, each with a unique condition, comprised the CCD ( Table 2). The aqueous portion of the mobile phase, 100 mL in volume, was made for each run. Once the pH was adjusted, the mobile phase was eluted at a 1.0 mL min ^-1^ rate in each run. TGC and its degradation products were measured at 275 nm. After collecting and analyzing all responses, models were created in design expert 13 to determine the interactive effects between the CQAs and the CMPs. The optimization process was designed to find the optimal Rs between the peaks of TGC and its degradation products without surrendering the other parameters that eventually determine the system's efficacy. Throughout this process, optimization was carried out both numerically and graphically.

**Table 2.** CCD for separating TGC and their degradation products under the studied chromatographic variables.

Runs	% Ethanol	pH	EDTA Conc	% TEA	Res 1	Res 2	Sym	T-plates	K factor	Run time
1	15	6.25	0.02	0.00	7.17	4.90	1.31	3303.45	4.05	5.06
2	10	5.00	0.01	0.10	1.02	1.61	1.37	2652.43	1.25	2.25
3	15	6.25	0.04	0.20	4.23	3.77	1.32	3254.47	2.76	3.77
4	20	5.00	0.01	0.30	0.40	2.64	1.08	2111.30	0.49	1.50
5	15	6.25	0.02	0.20	6.56	4.33	1.25	3449.69	3.77	4.78
6	10	5.00	0.01	0.30	1.65	2.00	1.50	2667.43	1.34	2.35
7	25	6.25	0.02	0.20	3.50	2.25	1.50	2000.88	1.45	3.00
8	10	5.00	0.03	0.30	3.26	1.53	1.24	2633.59	0.98	1.98
9	15	6.25	0.02	0.20	5.81	4.04	1.27	3117.93	3.49	4.50
10	15	3.75	0.02	0.20	0.30	0.30	1.20	1493.00	0.4	0.7
11	20	5.00	0.03	0.30	1.50	1.78	1.30	1763.00	0.50	1.50
12	20	7.5	0.01	0.30	10.00	3.28	1.50	3788.11	4.16	6.00
13	20	5.00	0.03	0.10	0.51	1.68	1.13	1780.37	0.32	1.32
14	15	6.25	0.02	0.20	7.08	4.50	1.23	3535.91	3.93	4.94
15	15	6.25	0.02	0.20	7.03	4.49	1.23	3477.36	3.89	4.89
16	15	6.25	0.00	0.20	9.09	4.28	1.21	3511.16	4.43	5.43
17	15	6.25	0.02	0.20	6.90	4.45	1.23	3556.05	3.81	4.82
18	20	5.00	0.01	0.10	0.42	1.71	0.70	2005.69	0.33	1.33
19	15	6.25	0.02	0.20	2.96	3.73	1.19	3395.66	3.57	4.57
20	10	5.00	0.03	0.10	0.50	1.57	1.25	2612.54	1.44	2.06

## Results

### Optimization

**Preliminary tests for screening**

The HPLC approach was selected to separate and quantify TGC and its degradation products. To establish a method for monitoring the stability of a product, the proposed chromatographic conditions must be developed and optimized. The impact of the relevant factors was screened using the preliminary experiments. TGC has a molecular weight of 585.65, log P values of -3.86, and pKa values of 3.19, 6.4, 7.54, and 9.14, respectively. ^6^^,^^50^ Water was selected as the appropriate solvent for TGC due to its high solubility. ^5^^,^^6^ All stock solutions in water were refrigerated for optimal drug stability in amber containers.

**Figure 2.**
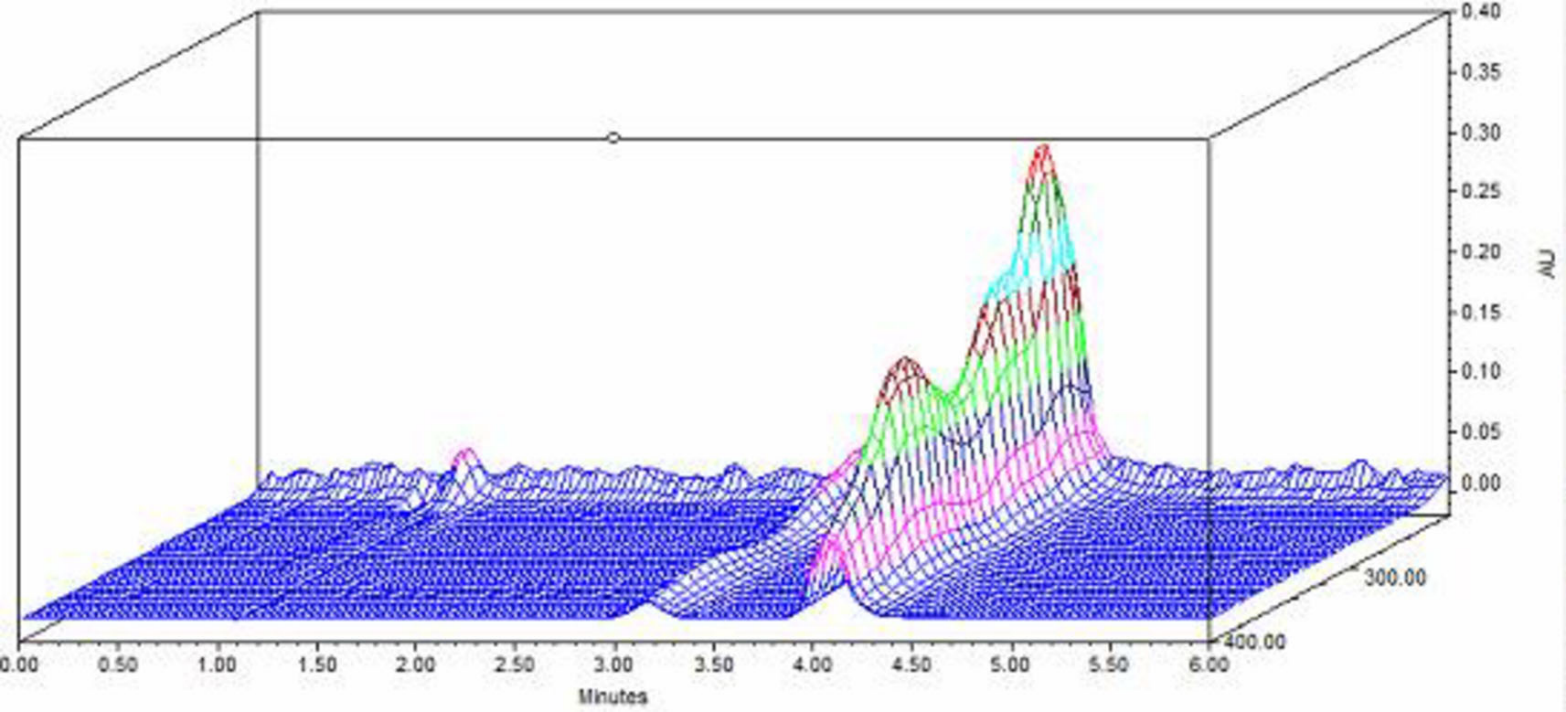
A 3D plot of the UV spectrum of TGC in distilled water.

Furthermore, an ultraviolet spectrophotometer scans 10 μg mL ^-1^ TGC solution to find appropriate wavelengths. The wavelength of the measurement must be 20 nanometers longer than the ultra-violet cutoff of the solvent. ^51^ The ultraviolet cutoff of ammonium acetate containing EDTA and TEA mixed with ethanol (used in reversed-phase liquid chromatography trials) was 210 nm. ^51^ Consequently, the UV detector was set at 275 nm for TGC detection because the latter wavelength would show acceptable sensitivity for the TGC peak with good peak shape and baseline in the HPLC chromatogram compared to a wavelength of 245 nm, so it was chosen for ED examinations ( Figure 2).

Two columns were employed to perform performance investigations. These columns were a VDSpher C18-E column (25*0.46 cm, 5 μm) from Nouryon (Sweden) and symmetry C18 Columns (10*0.46 cm, 3.5 μm) from Waters (Ireland). TGC is an antibiotic that resembles the chemical properties of tetracycline. TGC causes tailing peaks in a reversed-phase column by forming chelate complexes with metal ions and binding to the silanol group (Si-O-R). EDTA was added to the mobile phase to prevent forming of these chelate complexes as metals favor complexing with EDTA than TGC. Experiments demonstrated that the column, symmetry C18 Column, was more suitable because it had excellent chromatographic performance, ideal resolution, sensitivity, and peak symmetry while maintaining a shorter run time. A preliminary trial showed that mobile phase pH greater than 4 improves the peak shape of TGC but still needs improvement. As a result, some additives for mobile phases were tested for improving TGC peak shape and the resolution with its degradation products.

Higher pH values (4 or higher) combined with different concentrations of ethanol, EDTA, or TEA led to greater interference between the peaks of TGC and degradation products and a faster elution. These methods were developed to prevent adsorption on reversed-phase columns and chelate complexes formation. Therefore, the variables' minimum and maximum values were set at (10 and 20%), (5 and 7.5 mM), (10 and 40 mM), and (0.1 and 0.3%) for the percent of ethanol, mobile phase pH, EDTA molar concentration and percent of TEA, respectively. These values were utilized to figure out the possible range of the variables.

**RSM and CCD study**

Design Expert 13 software was used to make the ED, and RSM was used to explore the influence of the variables. ^52^ The model uses a CCD design, combining the independent factors in 20 different and five-centric runs. Our optimization study focused on the following factors: ethanol concentration, mobile phase pH, EDTA molar concentration, and percent of TEA. This study aimed to explore the sweet spot for these four factors. In addition, three distinct degrees of evaluation were carried out for each variable: -α, 0, and +α in Table 2.

As a result, the quadratic model can determine which parameters are most crucial. CCD is used for response modeling and optimization because it can comprehend the interactions between multiple variables. ^45^^–^^47^ The rotatable CCD was chosen because of its high consistency and relatively low variability. This experiment consisted of 20 randomized iterations that were used to assess the factor effects using (-α), (-1), (0), (+1), and (+α) values of examined variables.

**Response analysis and optimization**

The five selected responses were evaluated based on the criteria (ATP). Coded prediction models or equations allow one to compute the importance of various factors in light of the data at hand. ^46^ An artificial model selection approach used the Akaike information criterion (AIC) to improve accuracy. The P value is used in statistical analysis to determine the degree of significance ( Table 3). Compared to the threshold for statistical significance set at (P value = 0.05), all proposed models have minor P values (0.0001), indicating their significance. However, higher P values for the lack of fit indicate that they are not statistically significant (<0.05) ( Table 3). A positive coefficient in Table 3 positively impacts the corresponding response, whereas a negative coefficient indicates the opposite. ^52^ Therefore, other CCD aspects must be investigated to ensure model validity. Finally, strong linearity between the adjusted and expected regression coefficient (R2) was proved if the difference was smaller than 0.2 ( Table 3).

**Table 3.** Models for the CQAs and their attribute values are determined by minimizing the AICc in a backward approach.

Regression equation in terms of coded factors *	ANOVA p-value		Adjusted R ^2^	Predicted R ^2^	Adequate precision
	Model	Lack of fit			
ln (Res 1) = +1.70-0.2517A+1.70B-0.1774C-0.1621D+0.1240AB+0.2004AC-0.1726AD-0.3560BC-0.5922BD+0.3221CD	>0.0001	0.4881	0.9204	0.7736	16.1628
(Res 2) = +4.11-0.9781A+2.16B-0.1039C-0.4412D-1.13AB-0.0357AC+0.0732AD+0.0829BC-0.6254BD-0.1457CD	>0.0001	0.3111	0.9359	0.7906	20.1691
(sym) = +1.24-0.1340A+0.0350B+0.0247C-0.0324D+0.2781AB+0.1276AC+0.0541AD+0.0102BC-0.1158BD-0.0449CD	>0.0001	0.4035	0.9739	0.9296	38.6394
ln (plate) = +8.13-0.2665A+0.4150B-0.0.0187C-0.00148D-0.1044AB-0.0338AC+0.0033AD+0.0223BC-0.0077BD-0.0072CD	>0.0001	0.9847	0.9832	0.9800	34.1380
(k factor) = -3.65-1.22A+2.78B-3563C-0.3249D-0.8293AB+0.0519AC+0.0581AD-0.3044BC -0.3518BD-0.0356CD	>0.0001	0.0879	0.9728	0.8023	20.8663
(Run time) = +4.68-0.9073A+2.87B-0.3822C-0.2572D-0.5507AB+0.0849AC+0.0241AD-0.2954BC-0.3186BD-0.0031CD	>0.0001	0.3514	0.9864	0.9469	33.2867

* A=%Ethanol, B=Buffer pH, C=EDTA Conc, and D=%TEA.

There was also the signal-to-noise ratio, which had to be high and was approved if a value greater than 4 indicated appropriate precision. Based on previously existing data, it was possible to determine the relevance of the examined factors to the selected CQAs. The adequate precision allows for evaluating the signal-to-noise ratio, which should be greater than 4.0. Consequently, as shown in Table 3, the findings demonstrate that this model can explore the design space.

**Figure 3.**
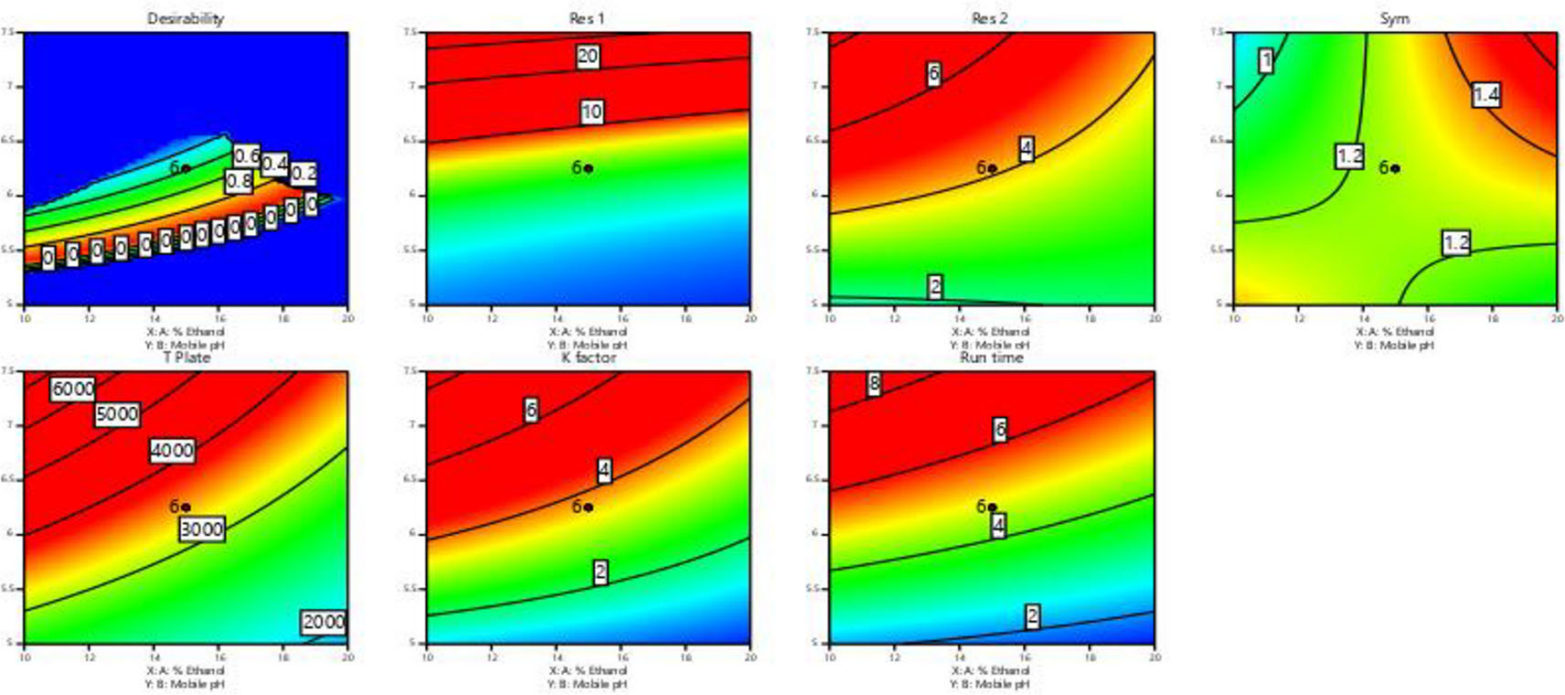
Contour Plots for the effect of dependent variables on all responses.

**Figure 4.**
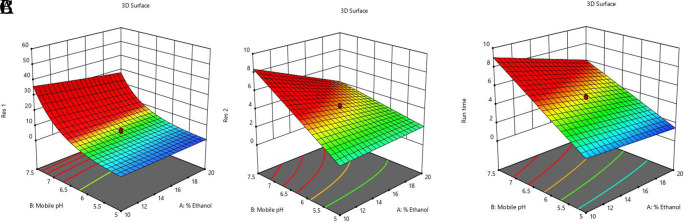
Plots of the 3D response surface for dependent variables, including (A) the resolution (Res 1), (B) (Res 2), and (C) run time.

**Figure 5.**
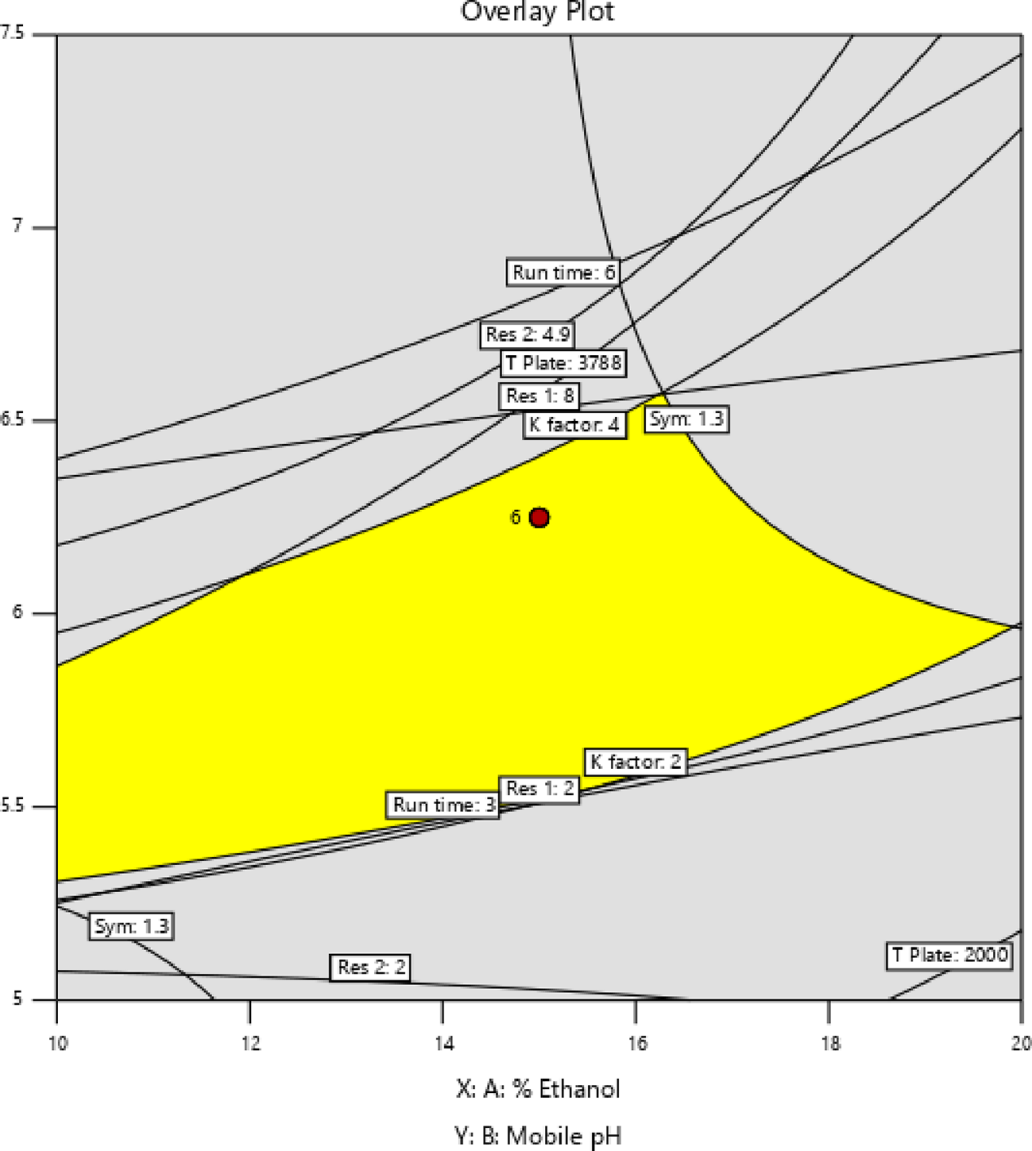
Overlay plot showing the sweet spot for the selected chromatographic parameters in response to acceptable factor settings.

In Figure 3 and Figure 4, contour and 3D surface plots depict the optimal responses against numerous combinations of essential variables. Finally, optimization was performed using both graphical and numerical approaches. In this stage, solutions are developed for the ideal chromatography settings with the best levels of desire by utilizing the limitations of the variables and their responses, as shown in Figure 5.

The primary objective of the optimizations was to attain a reasonable value for resolution (>2). Other parameters were also considered, including peak symmetry, theoretical plates, capacity factor, and the shortness of analysis time (run time). With graphical optimization, it's possible to put these limitations into practice. Finding the “sweet spot” (optimal value) between design space and robustness requires adjusting factor values (yellow area) ( Figure 5). However, results that fall into the gray area are generally undesirable. The pH varied between 6.0 and 6.4, the molar concentration of EDTA was between 10 and 40 mM, and the quantity of TEA was between 0.1 and 0.3%. The acceptable ranges are illustrated in Figure 5. The computed mean was inside the 95% confidence interval (CI) of the possible values when using the post-analysis procedure.

### Method validation

The method was validated by establishing its specificity, linearity, precision, accuracy, and robustness. The validation was done following the ICH requirements. ^48^

**Specificity**

**Figure 6.**
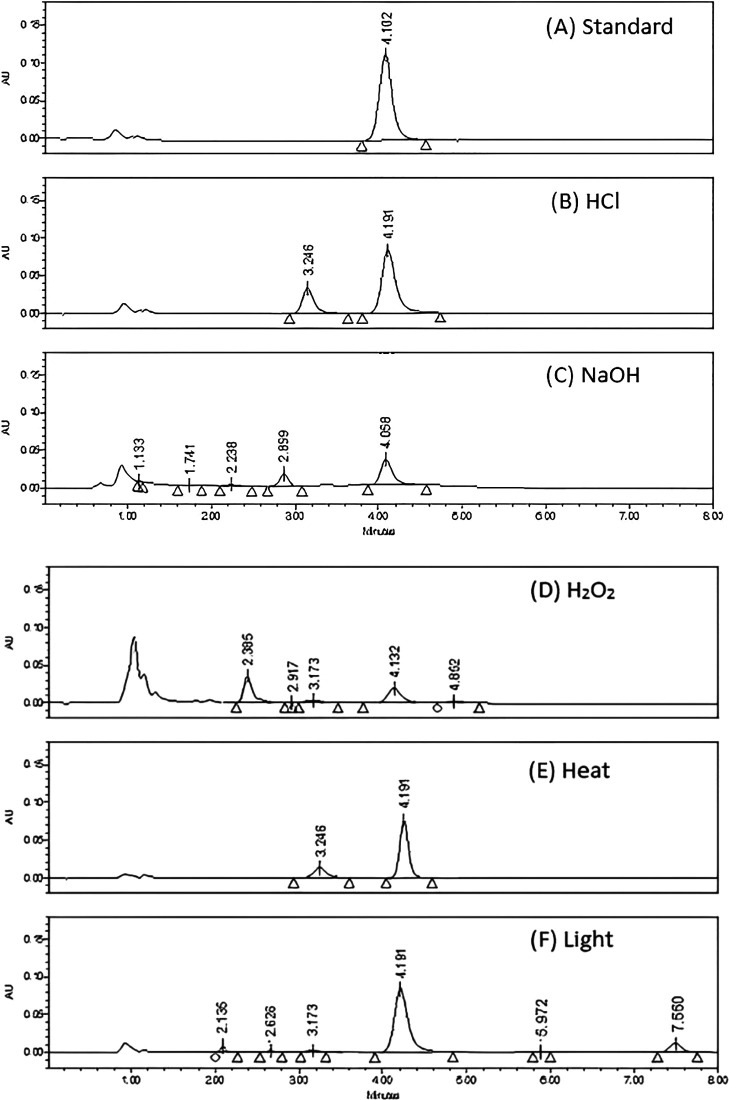
The proposed method is composed of a mobile phase containing ammonium acetate (50 mM), disodium edetate (20 mM), and triethylamine (0.2% v: v) (pH 6.25) - ethanol (85:15, v/v) running on Symmetry C18 column at a flow rate = 1 mL per minute.

Testing for excipient interference was performed by injecting a placebo (house-made solution of lyophilized powder excipient), and a known TGC concentration (40 μg mL ^-1^) was added to the placebo solution. The ability of the method to identify degradation products from the TGC peak is shown to have been successfully demonstrated by the excellent separation of the peaks, as shown in Figure 6.

**Linearity and range**

The linearity was confirmed by creating three different analytical graphs based on water, each with six different TGC levels in the 8–60 μg mL ^-1^ range. Before the injection of the solutions, the column was first allowed to become equilibrated for at least twenty minutes while the mobile phase was being passed via the system. As a point of reference, the area of peaks of the chromatograms and the concentrations of TGC were plotted on the analytical graph to construct the calibration graphs of TGC. Table 4 states that the results, calibration equation, and determination coefficient were calculated using regression analysis with the least-squares method.

**Table 4.** Analytical method parameters for the determination of TGC.

Parameter	Results
Linearity range	8-60 μg mL ^-1^
Slope	67478
Intercept	- 58699
Correlation coefficient	0.9999
LOD	0.5 μg mL ^-1^
LOQ	1.6 μg mL ^-1^
Resolution	3.54
Peak symmetry	1.19
Theoretical plate	3284.85
Capacity factor	3.42

**Precision**

The precision of the approach was assessed based on its repeatability and intermediate precision. Six determinations were performed on the same day and under the same conditions to test the repeatability using the same TGC concentration sample. Analyses performed on three separate days and by other analysts in the same laboratory helped determine the method's intermediate precision (between analysts).

**Accuracy**

The accuracy of the proposed method was assessed by adding 32, 40, and 48 μg mL ^-1^ to a sample solution equivalent to 80, 100, and 120% of the actual analytical level. As can be seen in Table 5, the percentage of sample recovery was utilized to calculate the percent bias (% error) between the recorded average concentrations and those that were added.

**Table 5.** Accuracy and precision of the proposed method for the determination of TGC.

	Labeled concentration μg mL ^-1^	Found concentration μg mL ^-1^	%Recovery	%RSD
**Accuracy**				
80%	32	31.8±0.02	99.47±0.10	0.19
100%	40	39.9±0.01	99.79±0.03	0.03
120%	48	48.0±0.01	99.98±0.03	0.03
**Repeatability**				
80%	32		99.56±0.09	0.09
100%	40		99.81±0.02	0.02
120%	48		100.22±0.44	0.05
**Intermediate**				
80%	32		99.47±0.14	0.14
100%	40		99.79±0.04	0.04
120%	48		99.98±0.10	0.10

R%: Recovery percentage.

SD: Standard deviation.

**LOD and LOQ**

The LOQ and LOD values were calculated using ICH, ^48^ the results are presented in Table 4. Calculating LOQ and LOD was accomplished by determining the standard deviation of the intercept and slope ratio over three different analytical graphs. These values were obtained using a linear regression model and multiplying the latter percentage by their respective detection and quantitation limit factors of 3.3 and 10. In addition, the LOQ was tested in an experiment.

**Robustness**

For routine analysis, the robustness of the suggested procedure can be judged by its capacity to withstand modest and purposeful changes in method parameters. For example, using the same samples (40 μg mL ^-1^ TGC) under five different analytical parameters, the robustness assessment of the chromatographic procedure for TGC quantification is illustrated in Table 6.

**Table 6.** Results of robustness studies for determination of TGC.

	Conditions	Changes	R%
1	**Proposed method** *	---	99.90%
2	Flow rate	0.9 mL min ^-1^	100.11%
3	Flow rate	1.1 mL min ^-1^	100.42%
4	Mobile phase pH	6.1	99.82%
5	Mobile phase pH	6.4	100.34%
6	% ETOH	13%	100.15%
7	% ETOH	17%	100.36%
8	Temperature	38°C	99.76%
9	Temperature	42°C	100.50%
10	UV detection	273 nm	100.36%
11	UV detection	275 nm	100.23%

* Proposed method is composed of a mobile phase containing ammonium acetate (0.05 M), disodium edetate (0.02 M), and triethylamine (0.2% v:v) (pH 6.25) - ethanol (85:15, v/v) running on a Symmetry C18 column at a flow rate = 1 mL min ^-1^. Detection was set at 275 nm, and the column temperature was kept at 40°C.

**System suitability test**

A system suitability test was also performed to verify the system's repeatability and resolution for the analysis. This test consisted of 6 replicate injections of a standard solution containing 40 μg mL ^-1^ TGC. Measurements were taken of peak symmetry, theoretical plates, and capacity factor. Observe and record the relative standard deviation of the peak area of injected standard samples and the retention times of all injections.

**Measurement of TGC in drug products**

After diluting the lyophilized TGC vial with water (40 μg mL ^-1^), filtering, and injecting in triplicate, the percent recoveries of the TGC against the pure compound were determined.

## Discussion

### Degradation kinetic study

ICH Q1A (R1) and Q1B recommendations were applied further to study the degradation of TGC under various stress conditions. ^10^^,^^49^

### Reactions of degradation

When no information regarding potential degradation products was available, stress degradation tests were employed to test the method's stability-indicating aspects. In addition, all stress degradation investigations generated probable degradation products. It was found that TGC was labile to all forced degradation conditions, including acidic, alkaline oxidative, thermal hydrolysis, and UV degradation. TGC showed significant decreases in the area under all conditions, ranging from 30% to 70% ( Table 7). Furthermore, each type of forced degradation scenario produced a distinct pattern of degradation products corresponding to specific degradation pathways. The TGC reference material and Tygacil ^®^ vial had nearly identical findings and practices. When the results of the proposed method were compared with other reported, it was found a similarity with the reported methods ^34^^,^^35^^,^^39^ that the oxidative and basic stress conditions have more effect on TGC than the acidic one.

**Table 7.** Percentage of degradation of TGC under forced degradation conditions.

Type	Proposed method	Reported methods			
		^34^	^35^	^39^	^40^
**Acidic degradation (60°C for 1 hour)**	30.2%	Slow	32% ^iii^	16%	10.1%
**Basic degradation (25°C for 2 hours)**	66.8%	Fast	28% ^iii^	35%	5.3%
**Oxidative degradation (25°C for 2 hours)**	78.5%	Very Fast	20% ^iii^	57%	3.6%
**Thermal degradation (60°C for 2 hours)**	11.8%	Fast	14%		31%
**Photo-degradation (RT for 24 hours)**	22.6%	Medium	8%		8.0%

^i^ Retention time of TGC is 4.1 min.

^ii^ deg stands for “degradation product.”

^iii^ Basic and oxidative stress conditions in method ^35^ were obtained in 10 and 15 min, but the acidic condition in 60 min.

### Greenness evaluation of the suggested method

To determine whether the proposed method was environmentally friendly, we used the analytical eco-scale to calculate the penalty points gained by each analysis step. A grade of over 75 suggests a special green assessment, while a degree of over 50 shows an acceptable green evaluation and a grade of 50 indicates inadequate green assessment. ^41^ Analytical procedures can be evaluated using the Green Analytical Procedure Index (GAPI), a new tool that assesses the environmental friendliness of the entire process. Using a unique symbol with five pentagrams, it is possible to use GAPI to analyze and quantify the ecological impact of each phase of an analytical technique. The colors green, yellow, plus red in the GAPI pentagram each stand for a different level of impact: low, middle, as well as high, respectively.

The National Environmental Methods Index (NEMI) labelling system is one of the earliest ways to gauge how environmentally friendly analytical processes are. There are four fields on the circular NEMI label. The fields are filled in green, representing a different stage in the provided analytical technique if certain requirements are met. The first criterion is that none of the substances must be on the potentially hazardous, persistent, and bio-accumulative chemicals list. The second requirement states that no substances used in the procedure may be on the D, F, P, or U lists of hazardous wastes. The pH of the sample must be between 2 and 12 to avoid creating a highly corrosive environment throughout the entire analytical procedure. ^41^

The number of Globally Harmonized System (GHS) of Classification and Labeling of Chemicals hazard pictograms was multiplied by the degree of hazard warning, which was multiplied by 1, and danger, which was multiplied by 2, to calculate the number of penalty counts that should be assigned to each reagent. Because the GHS risk pictograms are printed on the bottles of the reagents, it is easy to determine the level of danger connected with using the chemicals. ^42^^–^^44^^,^^53^ The interpretation of the GAPI pentagrams, NEMI, and Eco-scale assessment of the proposed chromatographic approach and its comparison with the reported methods ^33^^–^^37^^,^^39^^,^^40^ are described in Tables 8 and 9. The recommended procedure was more environmentally friendly than the reported methods. As a result, it can be used for routine analysis without causing harm to the environment.

**Table 8.** The penalty points of the proposed method are according to the analytical Eco-Scale.

Reagents/ instruments	The proposed method	Report methods						
		^33^	^34^	^35^	^36^	^37^	^39^	^40^
**Ethanol**	0	-	-	-	-	-	-	-
**Acetonitrile**	-	8	8	8	8	8	8	-
**Ammonium acetate buffer**	0	2	-	-	-	-	-	-
**Sodium acetate buffer**	-	-	0	-	-	-	-	-
**Acetic acid (glacial)**	2	-	-	2	-	-	-	-
**DMSO**	-	5	-	-	-	-	-	-
**EDTA**	3	-	-	-	3	-	-	-
**Methanol**	-	-	-	-	-	-	-	12
**Oxalic acid**	-	-	3	-	-	-	-	-
**Phosphate buffer**	-	-	2	-	2	2	-	-
**Triethylamine**	0	-	-	-	-	-	-	3
**Trifluoroacetic acid**	-	-	-	-	-	-	2	-
**HPLC instrument**	2	2	2	2	2	2	2	2
**Occupational hazard**	0	0	0	0	0	0	0	0
**Waste**	5	5	5	5	5	5	5	5
**Total Penalty points**	**12**	**22**	**20**	**17**	**20**	**17**	**17**	**22**
**Analytical Eco-Scale Total Score**	**88**	**78**	**80**	**83**	**88**	**83**	**83**	**78**

**Table 9.** The GAPI and NEMI systems evaluate the greenness of the proposed and reported methods.

Method	Test conditions/ Validation parameters	GAPI	NEMI
**The proposed** **method**	Waters HPLC instrument using A mobile phase of buffer solution (50 mM ammonium acetate, 20 mM disodium edetate, 0.2% triethylamine) and ethanol 85:15 (v:v) was used for the liquid chromatography. The column thermostat was maintained at 40°C. It was filtered with a membrane filter.	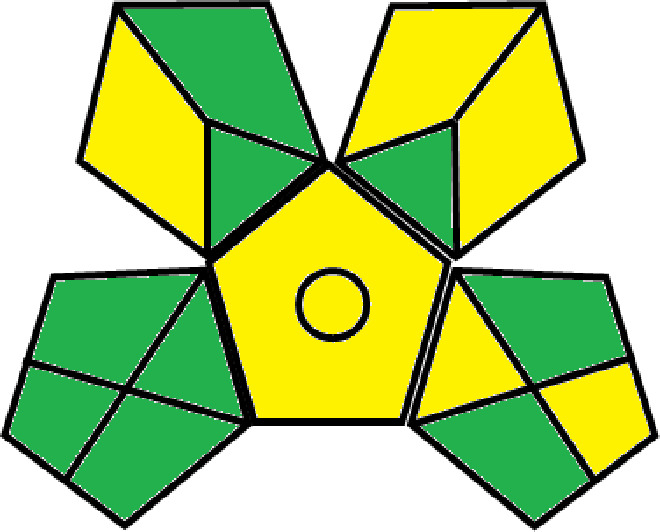	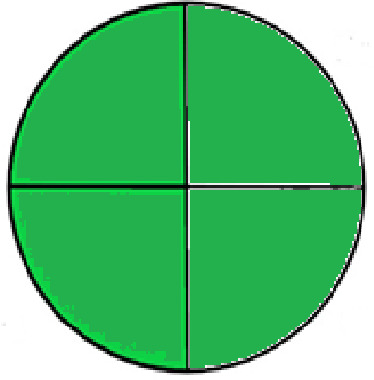
^33^	Waters HPLC instrument using Zorbax Eclipse plus C18 column with eluent-A: pH 6.50 buffer: acetonitrile: DMSO (90:5:5 %v/v/v) and eluent-B: pH 6.50 buffer: acetonitrile: DMSO (71:24:5 %v/v/v) as mobile phase at a 1.0 mL/min flow rate.	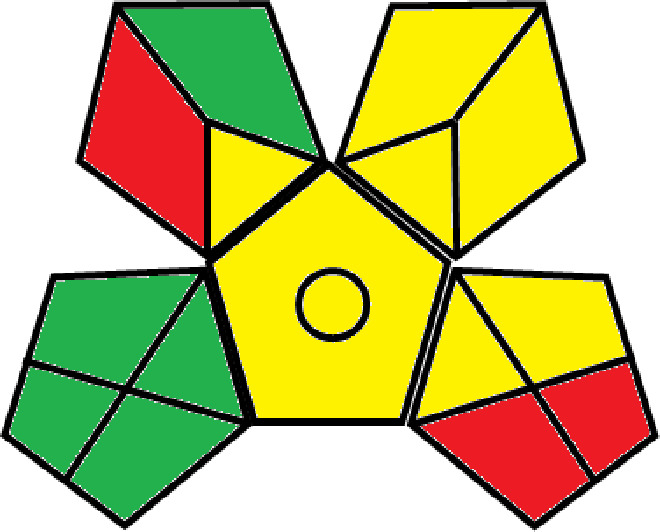	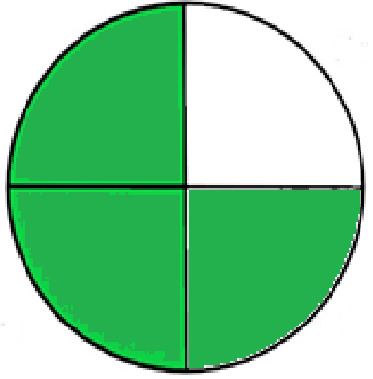
^34^	HPLC instrument using the mobile phase consisted of buffer containing sodium phosphate monobasic (0.015M) and oxalic acid (0.015M) (pH 7.0)–acetonitrile (75:25, v/v), run at a flow rate of 1.0 mL/min.	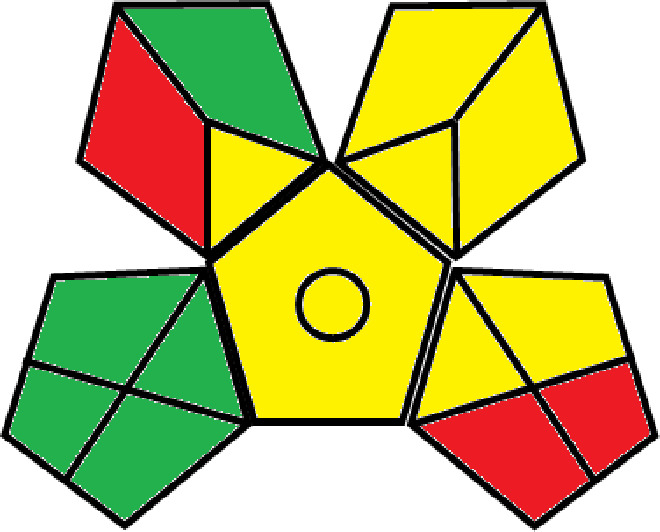	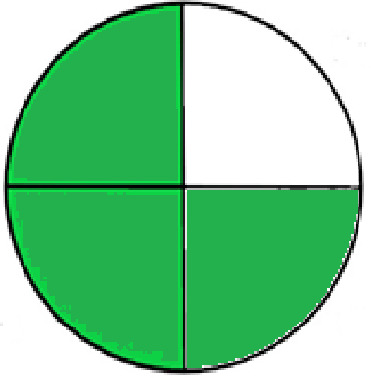
^35^	HPLC instrument using a reversible phase C18 column with a mobile phase consisting of a mixture of acetonitrile and acetic acid (0.1% aqueous solution, pH: 3.5) in the ratio of 20:80. Flow rate was 0.4 mL/min. Detection was carried out at 250 nm.	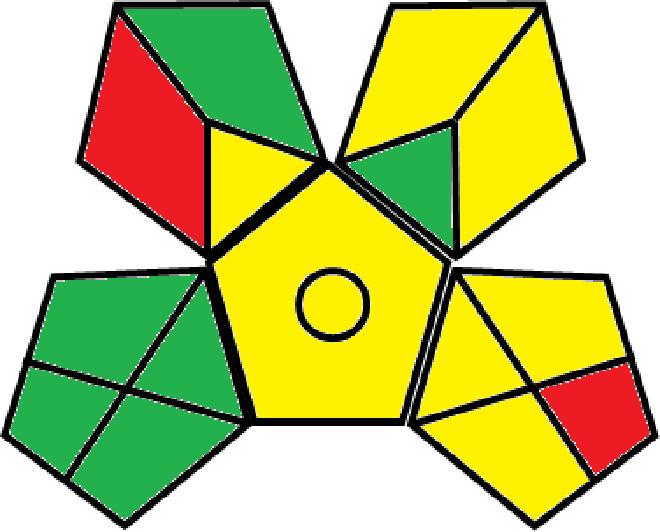	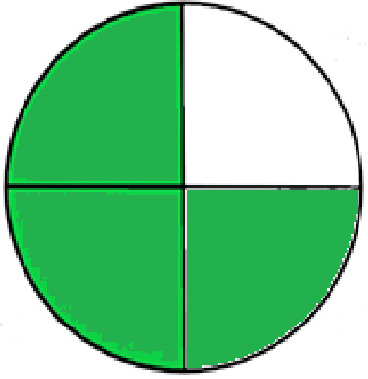
^36^	The analytical column was Thermo Acclaim™ 120 C18 with a volatile salt mobile phase at the flow rate of 1.5 mL min−1 (right pump). The mobile phase consisted of dipotassium hydrogen phosphate and EDTA solution adjusted to pH 6.4 with phosphoric acid)–acetonitrile (95:5) (A) pH 6.4 with phosphoric acid)–acetonitrile (50:50) (B).	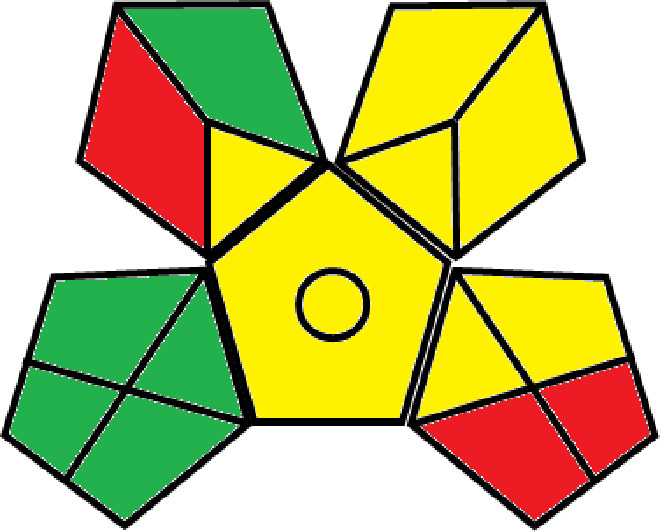	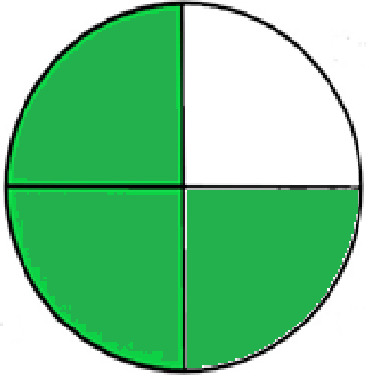
^37^	RP-HPLC method was carried out on a Kromasil ODS C-18 column using Buffer: Acetonitrile 83: 17 as mobile phase at a 1.2 mL/min flow rate.	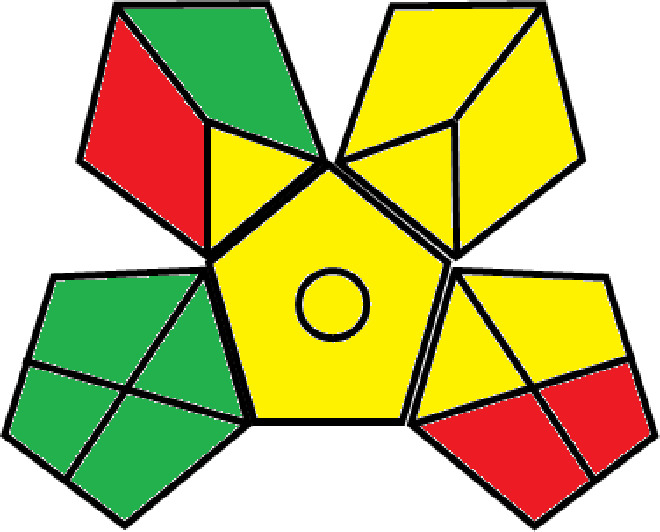	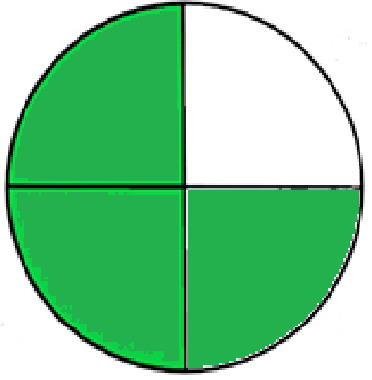
^39^	HPLC using a Kinetex C18 column. The mobile phase was composed of 0.1% trifluoroacetic acid (TFA) in water (A) and 0.1% TFA in acetonitrile B). The flow rate is 1 mL/min.	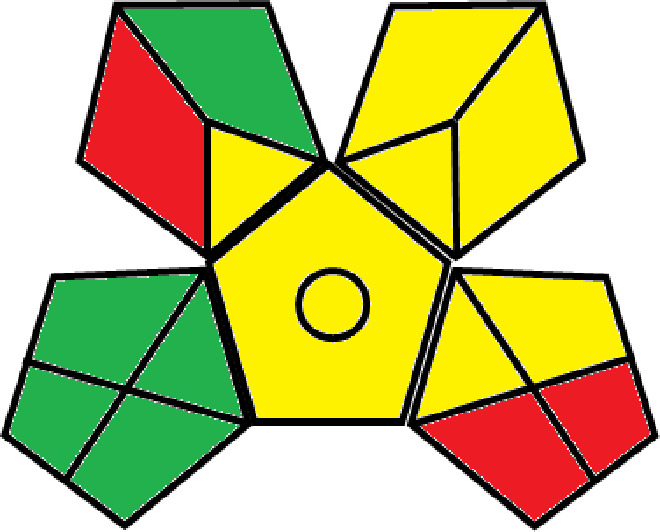	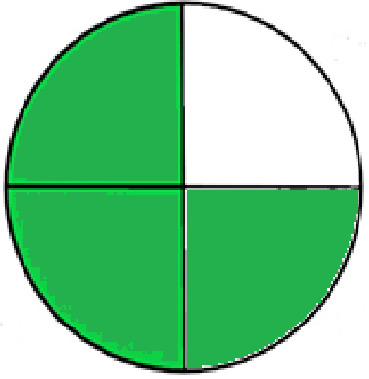
^40^	The stationary phase is Agilent ZORBAX Eclipse XDB column, Methanol and 10 mmol Triethylamine Buffer at pH 6.1 in the ratio of 75:25 (v/v). The flow rate is 1 mL/min.	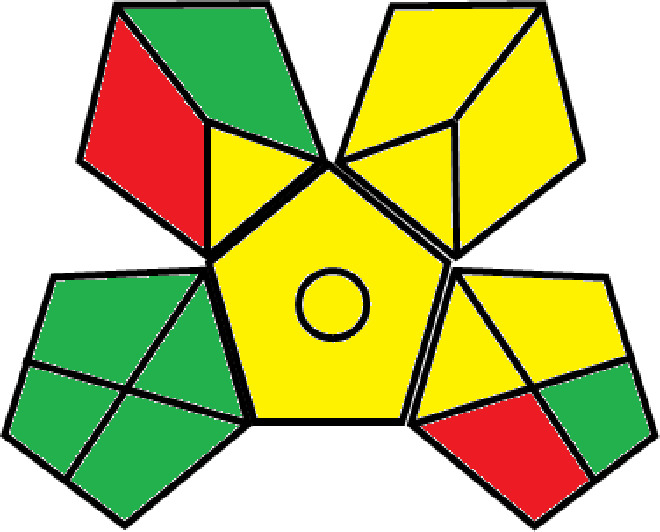	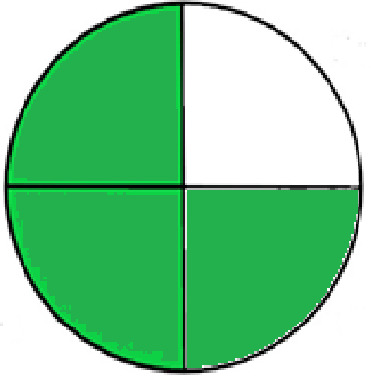

### Comparison with reported methods

Although the new method's success is achieving its goals, utilizing the GAC principles, and keeping a good resolution, fast analysis, and accurate results, comparing it with other reported methods is necessary. As discussed in the previous section, it is superior to other methods according to 3 greenness systems, GAPI, NEMI and Eco-scale in short-time analysis, as shown in Tables 8 and 9. The proposed method showed degradation rates similar to most reported methods, as shown in Table 7.

## Conclusion

In this research work, an ED approach for analytical method development consists of (i) developing a full grasp of the intended purpose, (ii) developing forecasting solutions, (iii) constructing an insightful system suitability solution that helps to identify modes of failure, and (iv) follows a design of experiments approach to method development. A central composite design was used to determine the impact of four chromatographic parameters on the chosen CQAs based on the risk assessment. The optimum analytical conditions were projected by a numerical optimization method. These conditions were located by flagging all parameters in an overlay plot. To study the main effects and interactions among different CMPs with the CAAs, 2D-contour plots and 3D-response surface plots were drawn.

The proposed method estimated the average amount of TGC in pharmaceutical products without interfering with the excipients. The process separates TGC from its degradation products rapidly. Eco-Scale and GAPI also demonstrated the method's greenness, which makes it more suitable for daily use.

## Data Availability

Figshare: Tig QbD chromatograms, https://doi.org/10.6084/m9.figshare.22153613.v1. ^54^ Data are available under the terms of the Creative Commons Attribution 4.0 International license (CC-BY 4.0).
