# Supplementary material for: Central composite design driven optimization of sustainable stability indicating HPLC method for the determination of Tigecycline and greenness assessment
Source: F1000Res. 2023 Aug 10;12:341. Originally published 2023 Mar 28. [Version 2] doi: 10.12688/f1000research.130861.2 (PMC10427804; doi:10.12688/f1000research.130861.2)
Supplement: Peer review history

# Reviewer report: Reviewer response for version 2

**DOI:** 10.5256/f1000research.152812.r195611

**Published:** 2023-08-18

**Authors:** Hala E. Zaazaa

**Affiliations:** 1 Analytical Chemistry Department, Faculty of Pharmacy, Cairo University, Giza, Giza Governorate, Egypt

I accept the manuscript. The authors respond correctly to all comments.

Is the work clearly and accurately presented and does it cite the current literature?

Yes

If applicable, is the statistical analysis and its interpretation appropriate?

I cannot comment. A qualified statistician is required.

Are all the source data underlying the results available to ensure full reproducibility?

Yes

Is the study design appropriate and is the work technically sound?

Yes

Are the conclusions drawn adequately supported by the results?

Partly

Are sufficient details of methods and analysis provided to allow replication by others?

Yes

Reviewer Expertise:

Pharmaceutical analytical Chemistry

I confirm that I have read this submission and believe that I have an appropriate level of expertise to confirm that it is of an acceptable scientific standard.

# Reviewer report: Reviewer response for version 2

**DOI:** 10.5256/f1000research.152812.r195613

**Published:** 2023-08-15

**Authors:** Ali Fouad

**Affiliations:** 1 Pharmaceutical Chemistry Department, Faculty of Pharmacy, Al-Azhar University, Nasr City, Cairo Governorate, Egypt

Regarding the revised manuscript, the authors addressed all the requested comments.

Is the work clearly and accurately presented and does it cite the current literature?

Yes

If applicable, is the statistical analysis and its interpretation appropriate?

Yes

Are all the source data underlying the results available to ensure full reproducibility?

Yes

Is the study design appropriate and is the work technically sound?

Yes

Are the conclusions drawn adequately supported by the results?

Yes

Are sufficient details of methods and analysis provided to allow replication by others?

Yes

Reviewer Expertise:

Medicinal chemistry, Pharmaceutical analysis

I confirm that I have read this submission and believe that I have an appropriate level of expertise to confirm that it is of an acceptable scientific standard.

# Reviewer report: Reviewer response for version 2

**DOI:** 10.5256/f1000research.152812.r195612

**Published:** 2023-08-15

**Authors:** Eman I. El-Kimary

**Affiliations:** 1 Department of Pharmaceutical Analytical Chemistry, Faculty of Pharmacy, Alexandria University, Alexandria, Alexandria Governorate, Egypt

I checked the revised version of the manuscript and the authors have responded appropriately to all the comments raised by the reviewer. The manuscript is now Approved for indexing in its current form.

Is the work clearly and accurately presented and does it cite the current literature?

Yes

If applicable, is the statistical analysis and its interpretation appropriate?

Yes

Are all the source data underlying the results available to ensure full reproducibility?

Yes

Is the study design appropriate and is the work technically sound?

Yes

Are the conclusions drawn adequately supported by the results?

Yes

Are sufficient details of methods and analysis provided to allow replication by others?

Yes

Reviewer Expertise:

Pharmaceutical Analytical Chemistry

I confirm that I have read this submission and believe that I have an appropriate level of expertise to confirm that it is of an acceptable scientific standard.

# Reviewer report: Reviewer response for version 1

**DOI:** 10.5256/f1000research.143649.r175968

**Published:** 2023-06-15

**Authors:** Hala E. Zaazaa

**Affiliations:** 1 Analytical Chemistry Department, Faculty of Pharmacy, Cairo University, Giza, Giza Governorate, Egypt

Greenness assessment and QbD are essential aspects during method development. There is some interest in publishing the manuscript which, however, it also has a number of significant shortcomings that should be addressed before the manuscript is acceptable for indexing:

- While authors summarize a comparison between their suggested method and the previously published ones, they missed this article ^1^ that was published in International Journal of Applied Pharmaceutics 2021. I ask the authors to add it illustrating the difference between their work and the aforementioned article.
- It was valuable that the authors summarize the difference between their suggested method and the previously published ones in a tabulated form and show the greenness assessment of each.
- It will be worthy to add other greenness assessment tools such as Eco-scale and NEMI.
- Please add a reference for the buffer preparation.
- Preparing reference substance solutions should not contain any results information so the statement "Water is considered the most suitable solvent for TGC because of its high solubility in water" should be omitted and transferred into the discussion section. The same for "An analytical method that successfully evaluates and assesses the active constituents without interfering with degradation products, process contaminants, inactive ingredients, or any other possible impurities is known as a stability-indicating procedure. ^5,10,47^" under Forced degradation tests of TGC.
- Linearity and range are separate items according to ICH guidelines. Also Precision and accuracy.
- No need for Figure 7.
- The precision should be expressed as repeatability and intermediate precision not (intra-day) and (inter-day) in Table 5.
- According to ICH guidelines, robustness should be tested by system suitability parameters especially resolution not only recover %.
- Where are the results of the System suitability test?
- Reference number of the proposed method (d) is missing in Table 8.
- No statistical analysis was illustrated by the authors.

Is the work clearly and accurately presented and does it cite the current literature?

Yes

If applicable, is the statistical analysis and its interpretation appropriate?

I cannot comment. A qualified statistician is required.

Are all the source data underlying the results available to ensure full reproducibility?

Yes

Is the study design appropriate and is the work technically sound?

Yes

Are the conclusions drawn adequately supported by the results?

Partly

Are sufficient details of methods and analysis provided to allow replication by others?

Yes

Reviewer Expertise:

Pharmaceutical analytical Chemistry

I confirm that I have read this submission and believe that I have an appropriate level of expertise to confirm that it is of an acceptable scientific standard, however I have significant reservations, as outlined above.

## References

[1] : SIMULTANEOUS DETERMINATION OF TIGECYCLINE AND ITS POTENTIAL IMPURITIES BY A STABILITY-INDICATING RP-HPLC-UV DETECTION TECHNIQUE. *International Journal of Applied Pharmaceutics* .2022; 10.22159/ijap.2022v14i1.41243 75-82 10.22159/ijap.2022v14i1.41243

# Response

**Published:** 2023-07-07

**Authors:** Hani Hafez

**Affiliations:** Al-Esraa University College, Iraq

**Third reviewer: (Prof. Dr. Hala E. Zaazaa)**

 Greenness assessment and QbD are essential aspects during method development. There is some interest in publishing the manuscript which; however, it also has a number of significant shortcomings that should be addressed before the manuscript is acceptable for indexing:

1. **While authors summarize a comparison between their suggested method and the previously published ones, they missed this article 1** **that was published in International Journal of Applied Pharmaceutics 2021. I ask the authors to add it illustrating the difference between their work and the aforementioned article.**
**Answer 1: **Thank you, the article was added. The comparison was created from 2 points: the degradation percentage (Table 7) and the greenness assessment (Tables 8 and 9).
2. **It was valuable that the authors summarize the difference between their suggested method and the previously published ones in a tabulated form and show the greenness assessment of each.**
**Answer 2: **Thank you **, **the comparison was created from 2 points: the degradation percentage (Table 7) and the greenness assessment (Tables 8 and 9).
3. **It will be worthy to add other greenness assessment tools such as Eco-scale and NEMI.**
**Answer 3: **Thank you, the eco-scale and NEMI were added.
4. **Please add a reference for the buffer preparation.**
**Answer 4: **Thank you **, **the trials in the development stage obtained the buffer preparation.
5. **Preparing reference substance solutions should not contain any results information so the statement "Water is considered the most suitable solvent for TGC because of its high solubility in water" should be omitted and transferred into the discussion section. The same for "An analytical method that successfully evaluates and assesses the active constituents without interfering with degradation products, process contaminants, inactive ingredients, or any other possible impurities is known as a stability-indicating procedure. ^5,10,47^" under Forced degradation tests of TGC**.
**Answer 5: **Thank you, the sentences were edited and transferred.
6. **Linearity and range are separate items according to ICH guidelines. Also precision and accuracy**.
**Answer 6: **Thank you for your valuable comment, It is correct, as you mentioned, but the linearity should be described in a certain range according to the Q2R1 (The linearity of an analytical procedure is its ability (within a given range) to obtain test results which are directly proportional to the concentration (amount) of analyte in the sample). So that we merge linearity and range in the same paragraph. Precision and accuracy were edited and separated
7. **No need for Figure 7.**
**Answer 7: **Thank you, it was removed
8. The precision should be expressed as repeatability and intermediate precision, not (intra-day) and (inter-day) in Table 5.
**Answer 8: **Thank you, it was done
9. According to ICH guidelines, robustness should be tested by system suitability parameters, especially resolution, not only recover %.
**Answer 9: **Thank you for your valuable comment; the robustness test was performed on authentic material without degradation products, but the resolution was considered in the development stage and it was a critical quality attribute (CQA) with targeted ATP is NLT 2  in quality by design approach to select the appropriate design space.
10. Where are the results of the System suitability test?
**Answer 10: **Thank you **, **the results of the System suitability test were added in table 1
11. Reference number of the proposed method (d) is missing in Table 8.
**Answer 11: **Thank you, the proposed method (d) is the new method of this paper.
12. No statistical analysis was illustrated by the authors.
**Answer 12: **Thank you,

# Reviewer report: Reviewer response for version 1

**DOI:** 10.5256/f1000research.143649.r175432

**Published:** 2023-06-14

**Authors:** Ali Fouad

**Affiliations:** 1 Pharmaceutical Chemistry Department, Faculty of Pharmacy, Al-Azhar University, Nasr City, Cairo Governorate, Egypt

The topic of this manuscript is interesting. However, the text is contains some inaccurate expressions as from the technical/scientific, as well as from the linguistic points of view.

 Below just a number of small changes to the article.

1. In introduction, paragraph three, LC determinations should be differentiated to HPLC And LC/MS/MS methods (ref. 15-26).
2. The chemical structure of TGC needs to be adjusted by chem-draw.
3. In page 4, optimized chromatographic conditions mobile phase might be written as 85:15 (v:v). Also, nm is more common than nanometre.
4. In page 5, Forced degradation tests of TGC, (neutral) should be removed.
5. In page 5, Acidic forced degradation was examined at 25 and 60°C while Alkaline forced degradation was examined at 25°C only. In table 7, the results were for acidic and alkaline at 25 and 60°C for each.
6. In table 1, what is QMTP? It is quality method target profile, it's used only abbreviated, and it should be illustrated.
7. CCD, ED, CQA, ATPs, CMPs and QMTP approaches were used in this manuscript, so, some details about each should be added in the introduction or in methodology.
8. Authors should go through the manuscript regarding English grammar and make some edits.

Overall, the article is useful, albeit there are some statements which are scientifically not supported by proper reference and some statements which need to be rephrased.

Is the work clearly and accurately presented and does it cite the current literature?

Yes

If applicable, is the statistical analysis and its interpretation appropriate?

Yes

Are all the source data underlying the results available to ensure full reproducibility?

Yes

Is the study design appropriate and is the work technically sound?

Yes

Are the conclusions drawn adequately supported by the results?

Yes

Are sufficient details of methods and analysis provided to allow replication by others?

Yes

Reviewer Expertise:

Medicinal chemistry, Pharmaceutical analysis

I confirm that I have read this submission and believe that I have an appropriate level of expertise to confirm that it is of an acceptable scientific standard, however I have significant reservations, as outlined above.

# Response

**Published:** 2023-07-07

**Authors:** Hani Hafez

**Affiliations:** Al-Esraa University College, Iraq

**Second reviewer: (Prof. Dr. Ali Fouad)**

 The topic of this manuscript is interesting. However, the text contains some inaccurate expressions from the technical/scientific, as well as from the linguistic points of view.

 Below are just several small changes to the article.

1. **In paragraph three's introduction, LC determinations should be differentiated into HPLC and LC/MS/MS methods (ref. 15-26).**
**Answer 1:** Thank you, the paragraph was edited.
2. **The chemical structure of TGC needs to be adjusted by chem-draw.**
**Answer 2:** Thank you, the chemical structure of TGC was adjusted by chem-draw
3. **In page 4, optimized chromatographic conditions mobile phase might be written as 85:15 (v:v). Also, nm is more common than nanometre.**
**Answer 3:** Thank you, the required modifications were done
4. **In page 5, Forced degradation tests of TGC, (neutral) should be removed.**
**Answer 4:** Thank you, (neutral) was changed to thermal.
5. **On page 5, Acidic forced degradation was examined at 25 and 60°C while Alkaline forced degradation was examined at 25°C only. In table 7, the results were for acidic and alkaline at 25 and 60°C for each.**
**Answer 5:** Thank you,  Table 7 was corrected
6. **In table 1, what is QMTP? It is quality method target profile, it's used only abbreviated, and it should be illustrated.**
**Answer 6:** Thank you, Table 1 was corrected and the QMTP was changed to ATP, as described above in Table 1. 
7. **CCD, ED, CQA, ATPs, CMPs and QMTP approaches were used in this manuscript, so, some details about each should be added in the introduction or in methodology.**
**Answer 7:** Thank you, the paragraph was edited to describe only the degradation in the solid state. 
8. **Authors should go through the manuscript regarding English grammar and make some edits.**
**Answer 8:** Thank you, the manuscript was revised and edited.
9. **Overall, the article is useful, albeit there are some statements which are scientifically not supported by proper reference and some statements which need to be rephrased.**
**Answer 9: **the article was grammarly re-checked and re-phrased

# Reviewer report: Reviewer response for version 1

**DOI:** 10.5256/f1000research.143649.r175969

**Published:** 2023-06-08

**Authors:** Eman I. El-Kimary

**Affiliations:** 1 Department of Pharmaceutical Analytical Chemistry, Faculty of Pharmacy, Alexandria University, Alexandria, Alexandria Governorate, Egypt

The manuscript describes a green stability indicating assay method for the determination of Tegicycline antibiotic in lyophilized powder with a full greenness assessment of the developed method. Response surface methodology using central composite design was applied for the optimization of experimental conditions. The method was fully validated according to the ICH guidelines. I have the following comments:

  

1. In the abstract, what is meant by (stable HPLC)? The whole abstract needs to be revised. In the results part of the abstract, please mention some analytical figures of merit. The conclusion part of the abstract also needs to be edited.
2. Please update the introduction part with previously published stability indicating chromatographic assays. Also, in the discussion part, compare the degradation results in the proposed method with those obtained in previously published stability indicating assays.
3. In page 3 2nd paragraph, please remove (which depicts the molecule's chemical structure). Also, please revise the sentence (The reported solubility of TGC was found in water) as it is not clear.
4. In materials and chemicals, please add the full name of QPS.
5. Under (Optimized chromatographic conditions), remove (which was the same.) at the end of this paragraph. Also, please write (centimetre, micrometres, degrees Celsius, etc) in symbols not in words. In general, please revise the symbols of units in the whole manuscript to be in accordance to the metric system.
6. Some titles need to be more concise. e.g. (Construction of calibration graphs for the linearity test) could be (Construction of calibration graphs), (Preparation of solution for accuracy and precision tests) could be (Accuracy and precision).
7. Under thermal forced degradation, Please clarify whether TGC was subjected to thermal degradation in the solid state, followed by thermal degradation of its solution in water or not as it is not clear.
8. Please write the full name of QMTP.
9. Under section Results, Optimization, Preliminary tests for screening, 2nd paragraph, please correct ammonium formate to ammonium acetate. Also, in this paragraph the choice of detection wavelength is not clear. Please discuss briefly the absorption characteristics of TGC and its effect on choosing the detection wavelength.
10. Why EDTA is added to the mobile phase?
11. The quality of figure 6 needs to be improved.
12. In table 5 and 6, please write %Recovery not R% and %RSD not RSD.

Is the work clearly and accurately presented and does it cite the current literature?

Yes

If applicable, is the statistical analysis and its interpretation appropriate?

Yes

Are all the source data underlying the results available to ensure full reproducibility?

Yes

Is the study design appropriate and is the work technically sound?

Yes

Are the conclusions drawn adequately supported by the results?

Yes

Are sufficient details of methods and analysis provided to allow replication by others?

Yes

Reviewer Expertise:

Pharmaceutical Analytical Chemistry

I confirm that I have read this submission and believe that I have an appropriate level of expertise to confirm that it is of an acceptable scientific standard, however I have significant reservations, as outlined above.

# Response

**Published:** 2023-07-07

**Authors:** Hani Hafez

**Affiliations:** Al-Esraa University College, Iraq

**First reviewer: (Prof. Dr. Eman I. El-Kimary)**

 The manuscript describes a green stability-indicating assay method for determining Tigecycline antibiotic in lyophilized powder with a full greenness assessment of the developed method. Response surface methodology using a central composite design was applied to optimize experimental conditions. The method was fully validated according to the ICH guidelines. I have the following comments:

1. **In the abstract, what is meant by (stable HPLC)? The whole abstract needs to be revised. In the results part of the abstract, please mention some analytical figures of merit. The conclusion part of the abstract also needs to be edited.**
**Answer 1:** Thank you for our valuable comment, (stable HPLC) has been changed to stability, indicating the HPLC method within the abstract. The results and conclusions parts have been edited to fulfill the statement to clarify the significance of the proposed study.
2. **Please update the introduction part with previously published stability indicating chromatographic assays. Also, in the discussion part, compare the degradation results in the proposed method with those obtained in previously published stability-indicating assays.**
**Answer 2:** Thank you for our valuable comment; the introduction was updated by adding new reported methods (Ref 40). The discussion part was also updated to include the required comparison.
3. **On page 3, 2nd paragraph, please remove (which depicts the molecule's chemical structure). Also, please revise the sentence (The reported solubility of TGC was found in water) as it is unclear.**
**Answer 3:** Thank you, the first sentence has been removed, and the second one has been edited and transferred to the discussion part. 
4. **In materials and chemicals, please add the full name of QPS.**
**Answer 4:** Thank you, the full name of QPS has been added.
5. **Under (Optimized chromatographic conditions), remove (which was the same.) at the end of this paragraph. Also, please write (centimetre, micrometres, degrees Celsius, etc) in symbols, not words. In general, please revise the symbols of units in the whole manuscript to be in accordance with the metric system.**
** Answer 5:** Thank you, the first sentence (which was the same) has been removed, and the other modifications were addressed. 
6. **Some titles need to be more concise. e.g. (Construction of calibration graphs for the linearity test) could be (Construction of calibration graphs), (Preparation of solution for accuracy and precision tests) could be (Accuracy and precision).**
**Answer 6:** Thank you, these titles were edited.
7. **Under thermal forced degradation, Please clarify whether TGC was subjected to thermal degradation in the solid state, followed by thermal degradation of its solution in water, as it is unclear.**
**Answer 7:** Thank you, the paragraph was edited to describe only the degradation in the solid state. 
8. **Please write the full name of QMTP.**
**Answer 8:** Thank you, the QMTP was changed to ATP, as described above in Table 1
9. **Under section Results, Optimization, Preliminary tests for screening, 2nd paragraph, please correct ammonium formate to ammonium acetate. Also, in this paragraph, the choice of detection wavelength is not clear. Please discuss briefly the absorption characteristics of TGC and its effect on choosing the detection wavelength.**
**Answer 9:** Thank you, the ammonium formate was corrected to ammonium acetate. The paragraph was also edited to present the merit of choosing 275 nm over 245 nm wavelength.
10. **Why is EDTA added to the mobile phase?**
**Answer 10:** Thank you, the following sentence was added under (EDTA was added to the mobile phase to prevent forming of these chelate complexes as metals favor complexing with EDTA than TGC.). It was added under Results, Optimization, Preliminary tests for screening, 3 ^rd^ paragraph.
11. **The quality of figure 6 needs to be improved** **.**
**Answer 11** **: **The quality of Figure 6 was improved.
12. **In table 5 and 6, please write %Recovery not R% and %RSD not RSD.**
**Answer 12: **Thank you, %Recovery and %RSD were added
